# Gut Microbiome Analysis Can Be Used as a Noninvasive Diagnostic Tool and Plays an Essential Role in the Onset of Membranous Nephropathy

**DOI:** 10.1002/advs.202201581

**Published:** 2022-08-17

**Authors:** Jin Shang, Yiding Zhang, Ruixue Guo, Wenli Liu, Jun Zhang, Ge Yan, Feng Wu, Wen Cui, Peipei Wang, Xuejun Zheng, Ting Wang, Yijun Dong, Jing Zhao, Li Wang, Jing Xiao, Zhanzheng Zhao

**Affiliations:** ^1^ Department of Nephrology The First Affiliated Hospital of Zhengzhou University Zhengzhou Henan 450052 China; ^2^ Zhengzhou University Zhengzhou Henan 450000 P. R. China; ^3^ Laboratory Animal Platform of Academy of Medical Sciences Zhengzhou University Zhengzhou Henan 450000 P. R. China; ^4^ Laboratory of Nephrology The First Affiliated Hospital of Zhengzhou University Zhengzhou Henan 450052 P. R. China; ^5^ Department of Clinical Laboratory Peking Union Medical College Hospital Peking Union Medical College & China Academy of Medical Science Beijing 100730 China; ^6^ Department of Nephrology the Third Affiliated Hospital of Sun Yat‐Sen University Guangzhou Guangdong 510630 China; ^7^ Biobank of The First Affiliated Hospital of Zhengzhou University Zhengzhou Henan 450052 P. R. China

**Keywords:** fecal microbiota transplant, gut microbiome, membranous nephropathy, non‐invasive diagnosis

## Abstract

Membranous nephropathy (MN) is a common cause of nephrotic syndrome. The aim is to establish a non‐invasive diagnostic model of MN using differential gut microbiome analysis, and to explore the relationship between the gut microbiome and MN pathogenesis in vivo. 825 fecal samples from MN patients and healthy participants are collected from multiple medical centers across China. Key operational taxonomic units (OTUs) obtained through 16S rRNA sequencing are used to establish a diagnostic model. A rat model of MN is developed to explore the relationship between the gut microbiome and the pathogenesis of MN. The diversity and richness of the gut microbiome are significantly lower in patients with MN than in healthy individuals. The diagnostic model based on seven OTUs achieves an excellent efficiency of 98.36% in the training group and also achieves high efficiency in cross‐regional cohorts. In MN rat model, gut microbiome elimination prevents model establishment, but fecal microbiome transplantation restores the phenotype of protein urine. Gut microbiome analysis can be used as a non‐invasive tool for MN diagnosis. The onset of MN depends on the presence of naturally colonized microbiome. Early intervention in the gut microbiome may help reduce urinary protein level in MN.

## Graphical abstract

In this study, specific changes in gut microbial composition are found in patients with membranous nephropathy (MN). Key bacterial taxa analysis can be used to non‐invasively diagnose MN. The onset of MN depends on the existence of the natural gut microbiome. These findings may be used to provide novel targets for preventing and diagnosing MN.

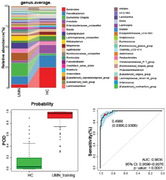

## 1 Introduction

Membranous nephropathy (MN) often causes nephrotic syndrome, and the condition is prevalent worldwide.^[^ ^1^, ^2^ ^]^ Several diagnostic tests, including M‐type phospholipase A2 receptor (PLA2R) and thrombospondin type 1 domain‐containing 7A, can be used for its diagnosis,^[^ ^3^, ^4^ ^]^ however, their sensitivity and specificity are not sufficiently high. Therefore, renal biopsies are still necessary to confirm the diagnosis, especially for PLA2R‐negative MN. The prognosis of MN is not optimistic, as 6–25% of MN patients fail to achieve clinical remission even after a series of treatments.^[^ ^5^, ^6^ ^]^ Those who achieve remission still have a relapse rate of up to 43–47%.^[^ ^5^, ^6^ ^]^ Therefore, novel and effective methods for early MN diagnosis and treatment are urgently needed.

The gut microbiome is linked to the development of many diseases, such as diabetes, systemic lupus erythematosus, and hyperthyroidism.^[^ ^7^, ^8^, ^9^, ^10^ ^]^ Despite the spatial discrepancy between the gut and kidney, tests to monitor specific changes in microbiome composition have been developed as non‐invasive diagnostic tools for different diseases.^[^ ^11^, ^12^ ^]^ This “invisible” connection is called gut–renal axis.^[^ ^13^ ^]^ Previous studies have compared the constitutional differences in the gut microbiome in MN with other primary or secondary kidney diseases.^[^ ^14^, ^15^ ^]^ However, the characteristic changes of gut microbes at different MN stages and their cause‐effect relationships remain unknown.

The aim of our study was to establish a non‐invasive model for MN diagnosis and to explore the key bacterial groups in MN. We also attempted to explain the causal relationship between the pathogenesis of MN and changes in the gut microbiome, which may provide new insights for the prevention and treatment of MN.

## 2 Results

**Figure 1:**
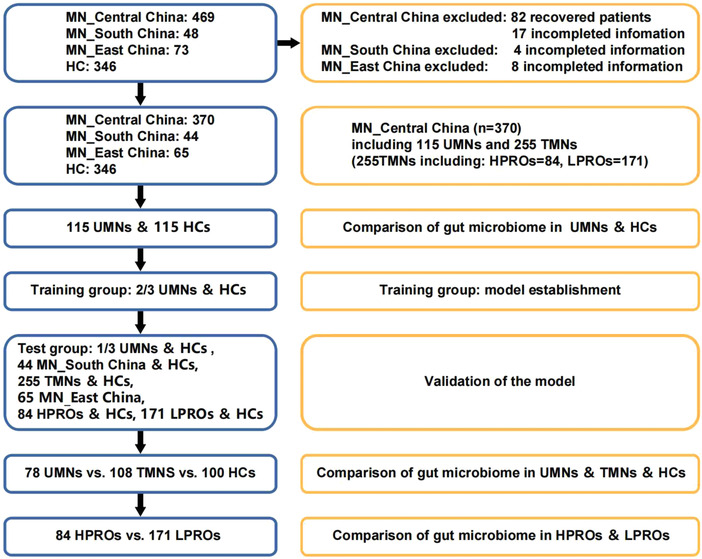
Study design and flow diagram: the profile of inclusion, exclusion, and grouping of patients. Following rigorous inclusion and exclusion criteria, a total of 825 fecal samples were collected including 370 samples of MN patients from Central China, 65 samples of MN patients from East China, 44 samples of MN patients from South China, and 346 samples of HCs from Central China. Gut microbiome was compared. Then 115 UMNs and matched 115 HCs were randomly divided into training group and test group to develop and validate diagnostic model based on key OTUs. Samples from different areas of China were included as cross regional cohort. Patients were divided further into different subgroups to test the influence of drugs and disease severity on intestinal flora. MN, Membranous nephropathy; UMN, untreated membranous nephropathy; TMN treated membranous nephropathy; HC healthy control; HPRO, high urine protein; LPRO, low urine protein.

Our study was designed based on the principles of prospective specimen collection and retrospective blinded evaluation.^[^ ^16^ ^]^ A total of 825 fecal samples from MN patients and healthy controls (HCs) were utilized in the 16S rRNA analysis. The study design and process are illustrated in **Figure** **1**. We attempted to establish and expand the scope of application of a diagnostic model for untreated MN (UMN). Therefore, we validated the diagnostic model in patients with MN different at differing states and geographical regions.

### 2.1 Gut Microbiome Profiles Were Significantly Altered in UMN Patients

**Figure 2:**
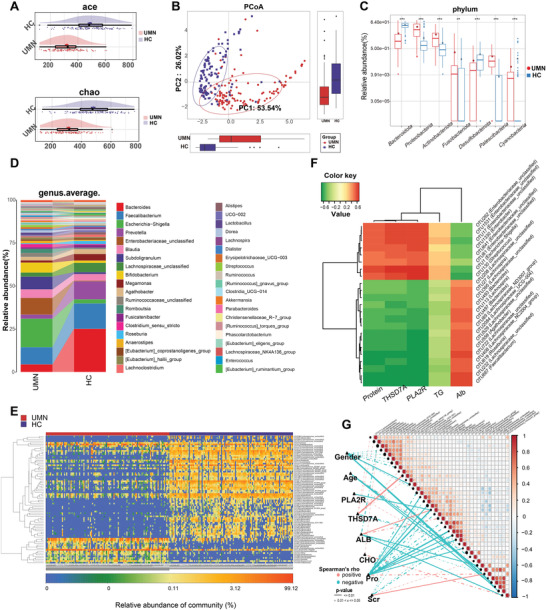
The composition and alteration of gut microbiota in UMNs (*n* = 115) and HCs (*n* = 115). A) Cloudplot showed *α*‐diversity in UMN group and HC group by ace index and chao index (*p* < 0.001). B) PCoA analysis showed visualized *β*‐diversity by unweighted UniFrac algorithm along PC1 and PC2. C) Wilcoxon rank sum test showed the distribution of gut microbiome in UMNs and HCs at phylum level. D) Average compositions and relative abundances of the bacterial communities in UMNs and HCs at genus level (Only the 40 genera with the highest relative abundance were listed). E) Heatmap showed the similarity and difference of key OTUs selected by random forest model in each participant of UMNs and HCs. F) Spearman correlation analysis showed the correlation between different OTUs and clinical indicators in heatmap (all *p* < 0.001). G) Spearman correlation analysis was used to analyze the degree of correlation between key OTUs and clinical indicators (Solid line, *p* < 0.01, dotted line, 0.01< *p* < = 0.05). OTU, operational taxonomic units; PCoA, principal coordinate analysis; POD, possibility of disease; PLA2R, phospholipase A2 receptor, PLA2R; THSD7A, thrombospondin type‐1 domain‐containing 7A; ALB, albumin; CHO, cholesterol; Pro, urine protein; Scr, serum creatinine; * *p* < 0.05; **p < 0.01, ***p < 0.001.

We compared the composition and alterations of the gut microbiome in 115 UMNs and 115 age and gender matched HCs. The baseline characteristics are shown in Table S1, Supporting Information. The results showed that the microbial composition was altered in UMNs compared to HCs, characterized by decrease in diversity and observed operational taxonomic units (OTUs) of the microbiome (**Figure** **2**A and Figure S1A–F, Supporting Information). Principal coordinate analysis (PCoA) diagrams also suggested a significant difference in microbial composition between the two groups (Figure 2B and Figure S1G,H, Supporting Information).

At the phylum level, the relative abundance of *Proteobacteria* and *Actinobacteria* were significantly higher, while the relative abundance levels of *Bacteroidota* were lower in UMNs compared to HCs (Figure 2C and Figure S2A–C, Supporting Information). At the genus level, *Bacteroides*, *Prevotella*, *Lachnospiraceae_unclassified*, and *Megamonas* were significantly lower in UMNs compared with HCs. However, the relative abundance level of *Escherichia−Shigella*, *Subdoligranulum*, *Bifidobacterium*, and *Enterobacteriaceae_unclassified* were higher in UMNs versus matched HCs (Figure 2D and Figure S2D, Supporting Information). These obvious changed bacteria taxa are likely to be related to the onset of MN. The heatmap also showed significant microbial differences in the key OTUs (Figure 2E).

Spearman correlation analysis showed a strong correlation between laboratory examination and gut microbiome features in MN (Figure 2F,G). Alteration in the predicted metabolic pathways of the gut microbiome (e.g., increased osmoprotectant transport) were also found in the UMNs (Figure S2E,F, Supporting Information). These results indicate that the gut microbiome composition and function were significantly altered in patients with UMN.

### 2.2 Establishment and Validation of the Diagnostic Model

**Figure 3:**
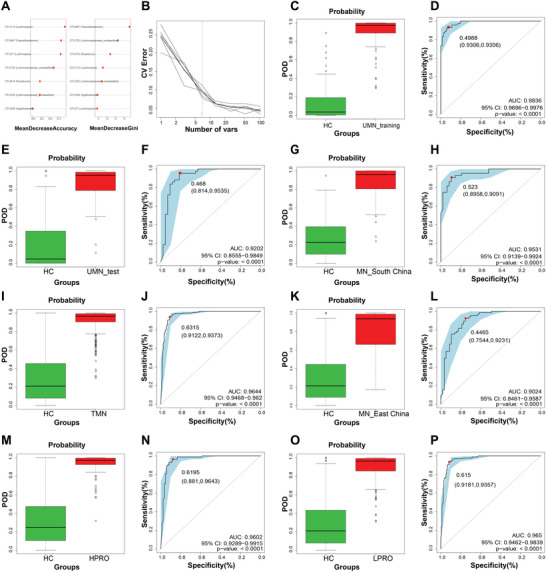
Development and validation of diagnostic models. A) Mean decrease accuracy and mean decrease gini showed the contribution values of 7 selected OTUs in the diagnostic model. B) Random forest model showed the optimal number of vars is 7. C) Diagrams of POD value based on microbial markers showed significant difference between UMNs (*n* = 72) and HCs (*n* = 72) in training group. D) ROC curve based on obtained microbial markers showed discrimination rate in training group. E) Diagrams of POD value based on microbial markers in test group (UMN_test = 43, HC = 43). F) ROC curve showed discrimination rate in test group. G) Diagrams of POD value of the validation cohort from South China (MN_South China = 44, HC = 48). H) ROC curve of the validation cohort from South China. I) Diagrams of POD value in TMNs (*n* = 255) and HCs (*n* = 205). J) ROC curve showed discrimination rate in TMNs and HCs. K) Diagrams of POD value in the validation cohort from East China (MN_East China = 65, HC = 57). L) ROC curve showed discrimination rate in the validation cohort from East China. M) Diagrams of POD value in HPROs (*n* = 84) and HCs (*n* = 84). N) ROC curve showed discrimination rate in HPROs and HCs. O) Diagrams of POD value showing difference between LPROs (*n* = 171) and HCs (*n* = 171). P) ROC curve showing discrimination rate in LPROs and HCs. POD, possibility of disease; CV, coefficient of variation; ROC, receiving operational curve.

To explore whether the gut flora could be used to help diagnose MN patients, 115 UMN patients and 115 matched HCs were randomly divided into a training group (UMN = 72, HC = 72) and a test group (UMN = 43, HC = 43). The baseline characteristics of the two cohorts are shown in Tables S2 and S3, Supporting Information. By means of fivefold cross‐validation of the random forest model, we obtained seven crucial OTUs, namely OTU27 (*Lachnospira*), OTU113 (*Lachnospira*), OTU232 (*Lachnospiraceae_unclassified*), OTU559 (*Agathobacter*), OTU619 (*Roseburia*), OTU667 (*Faecalibacterium*), and OTU722 (*Lachnospiraceae_unclassified*) to establish a diagnostic model (**Figure** **3**A,B). The possibility of disease (POD) and receiver operating characteristic (ROC) curve showed that the model had high efficiency with an area under curve (AUC) of 98.36% (cut‐off value, 0.4988; sensitivity, 0.9306; specificity, 0.9306) in the training group (Figure 3C,D). The test group also showed a high AUC of 92.02% (cut‐off value: 0.468; sensitivity: 0.9535; specificity: 0.814, Figure 3E,F). In the cross‐regional validation phase, composed of 44 UMN from South China and 48 matched HCs, the model showed good discriminative efficiency, with an AUC of 95.31% (cut‐off value, 0.523; sensitivity, 0.9091, specificity, 0.8958, Figure 3G,H).

Various medications can influence the gut microbiome in other systemic diseases.^[^ ^17^, ^18^ ^]^ In this study, we investigated whether the model could be used for treated membranous nephropathies (TMNs). The results showed that POD value level was markedly higher in TMNs than that in HCs, with an AUC of 96.44% (cut‐off value, 0.6315; sensitivity, 0.9373; specificity, 0.9122, Figure 3I,J). A regional cohort of 65 MN patients (treated or untreated) from East China and 57 matched HCs showed that the AUC was as high as 90.24% (cut‐off value, 0.4465; sensitivity, 0.9231; specificity, 0.7544, Figure 3K,L). These results suggest that medication had little impact on model efficiency, thus, it could be widely used in patients with MN, whether under medication or not.

To further evaluate its efficiency in different disease states, we divided TMNs into high protein group (HPRO, *n* = 84, urine protein≧3.5 g/24 h) and low protein group (LPRO, *n* = 171, urine protein < 3.5 g/24 h). After matching equal HCs, these two cohorts were recruited into the diagnostic model. The results showed that the AUCs were 96.02% (cut‐off value, 0.6195; sensitivity, 0.9643; specificity, 0.881) in the HPRO group (Figure 3M,N) and 96.5% (cut‐off value, 0.615; sensitivity, 0.9357; specificity, 0.9181) in the LPRO group (Figure 3O,P), indicating that the model was also suitable across different MN states.

### 2.3 Medication of MN Did Not Influence Gut Microbiome

To further explore the influence of medication on the gut microbiome, we compared 78 UMNs, 108 TMNs, and 100 HCs based on matched age and sex. Urine protein, serum creatinine, albumin, triglyceride, and total cholesterol levels were also matched in these UMNs and TMNs. The clinical data of the study are provided in Table S4, Supporting Information.

**Figure 4:**
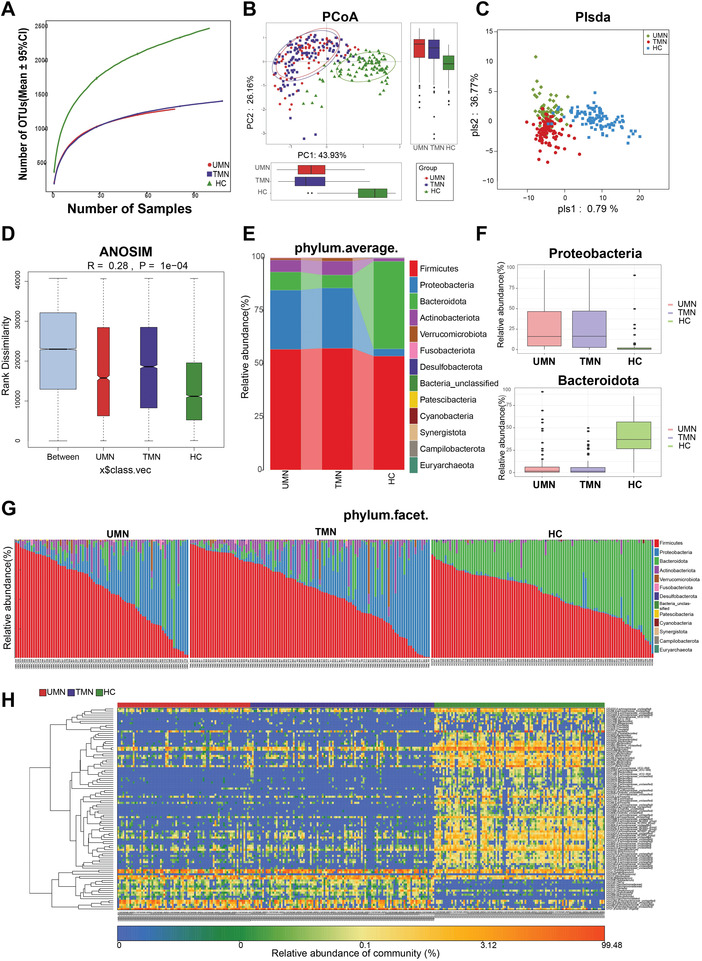
The composition and alteration of gut microbiota in UMNs (*n* = 78), TMNs (*n* = 108) and HCs (*n* = 100). A) Rarefaction curve showed the relationship between the number of observed OTUs and number of samples among three groups. B) PCoA analysis showed visualized *β*‐diversity of TMNs, UMNs, and HCs by unweighted UniFrac algorithm along PC1 and PC2. C) Plsda axis showed the different compositions among HCs, UMNs, and TMNs. D) ANOSIM analysis among three groups (*p* < 0.001). E) Average compositions and relative abundances of the bacterial communities among three groups at phylum level. F) The relative abundance of *Proteobacteria* increased while *Bacteroidota* decreased significantly both in UMNs and TMNs compared with HCs (*p* < 0.001). G) Average compositions and relative abundances of the bacterial communities of each sample in three groups at phylum level. H) Heatmap showed relative abundance of key OTUs selected by random forest model were more similar between UMNs and TMNs than HCs. ANOSIM, analysis of similarities; Plsda, Partial least squares discrimination analysis.

The observed OTUs were lower in both UMNs and TMNs compared with HCs (**Figure** **4**A). PCoA showed that the gut microbiome compositions were similar between UMNs and TMNs, but quite different from those of HCs (Figure 4B, Figure S3A,B, Supporting Information). Similar results were obtained by partial least squares‐discriminant analysis (PLS‐DA) and analysis of similarity (ANOSIM) (Figure 4C,D).

The analysis of relative abundance also suggested similar changes in the gut microbiome in UMNs and TMNs compared with HCs. The microbial composition at the phylum level showed that the relative abundance of *Proteobacteria* was higher, while that of *Bacteroidota* was significantly lower in both UMNs and TMNs compared with those in HCs (Figure 4E–G and Figure S3B, Supporting Information). A similar pattern was obtained at the genus level (Figure S3C, Supporting Information), suggesting synchronized microbial changes in UMNs and TMNs. The Venn diagram and heatmap revealed that UMNs and TMNs were more similar to each other than to HCs (Figure 4H and Figure S3D, Supporting Information). The Kyoto Encyclopedia of Genes and Genomes (KEGG) pathways also showed great differences between UMNs or TMNs and HCs (Figure S3E,F, Supporting Information). The specific functional pathway changed significantly, including an increase in human diseases in both UMNs and TMNs, which may be related to the pathogenesis of MN. These findings were quite different from our previous expectation, since the microbiome in MN does not seem to be significantly affected by medication.

### 2.4 The Changes of Gut Microbiome Remained Stable across Different Disease Status of MN

**Figure 5:**
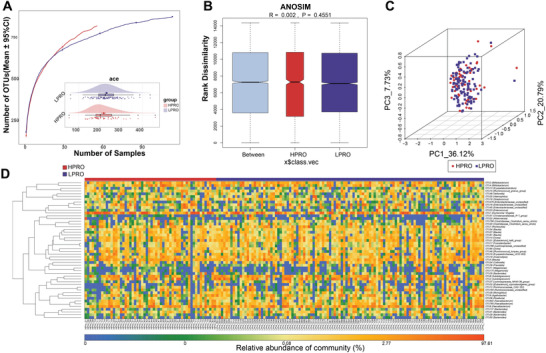
Comparison of gut microbiome between HPROs (*n* = 56) and LPROs (*n* = 114) in discovery phase. A) Rarefaction curve showed the relationship between number of observed OTUs and number of samples between two groups. The cloudpolt showing *α*‐diversity calculated by ace indexed was similar between two groups (*p* = 0.216). B) ANOSIM showed there was no difference between groups or within groups in HPROs and LPROs (*p* = 0.4551). C) PCoA analysis showed visualized *β*‐diversity of HPRO group and HC group by unweighted UniFrac algorithm. D) Heatmap showed the similarity and difference of top 50 most abundant OTUs in each participant of HPROs and LPROs.

A total of 255 patients with TMN were divided into HPROs (urine protein ≥3.5 g/24 h) and LPROs (urine protein < 3.5 g/24 h) according to their levels (clinical data are shown in Table S5, Supporting Information). To explore whether the gut microbiome could be used to distinguish the different states of MN patients, we further divided these individuals into a discovery (HPRO = 56, LPRO = 114) and a validation (HPRO = 28 LPRO = 57) phase. The clinical features of the two phases were shown in Tables S6 and S7, Supporting Information. We compared the gut microbiota composition between two groups in discovery phase. Interestingly, we found similar results in these two groups, including the observed OTUs and diversity (**Figure** **5**A). ANOSIM and PCoA also suggested that the gut microbiome of HPROs resembled that of LPROs (Figure 5B,C and Figure S4A–C, Supporting Information). The Venn diagram showed that 797 out of 897 OTUs were shared between both groups (Figure S4D, Supporting Information) and the heatmap also showed a great similarity of key OTUs (Figure 5E). It was difficult to find the differences in the relative abundances of microbial composition at the phylum or genus level (Figure S4E,F, Supporting Information). With fivefold cross‐validation of random forest model, eight key OTUs were obtained for the establishment of a new model. However, the model did not suggest a good discriminative ability (Figure S4G–J, Supporting Information). These results imply that changes in the gut microbiome are not associated with MN states.

### 2.5 Successful Construction of MN Rat Model Depends Largely on Gut Microbiome

**Figure 6:**
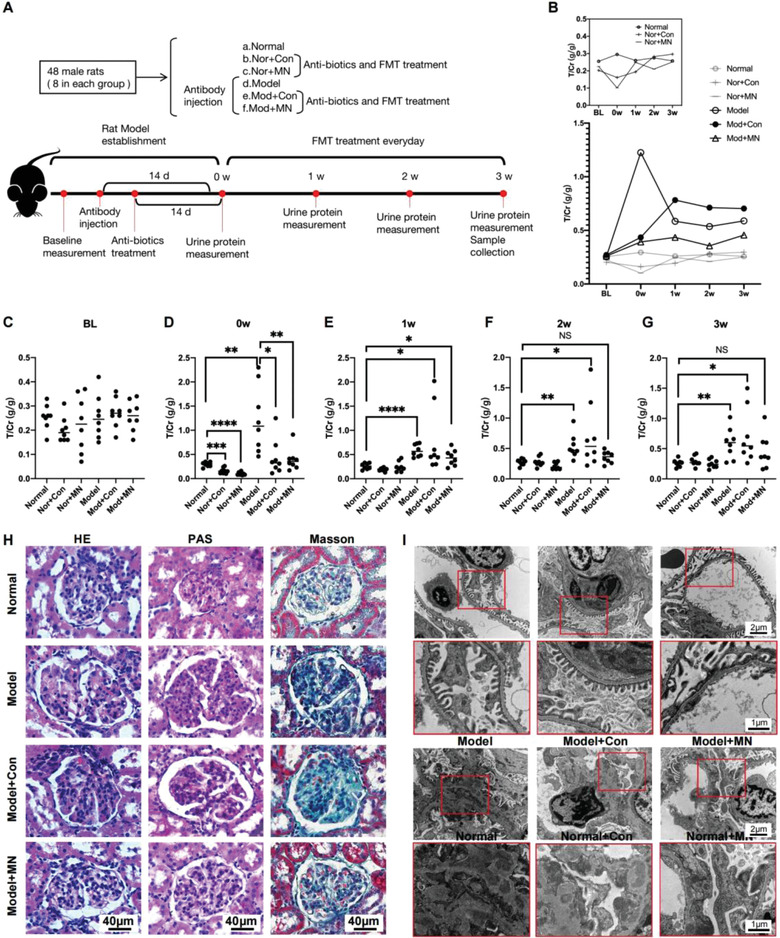
Clinical features and pathological changes in MN rat model (*n* = 8 in each group). A) Flow chart of rat experiment. B) Line chart showed the alteration of T/Cr in 6 groups from baseline to 3 weeks after FMT. C) Scatter plot of rat urine protein at baseline. D) Scatter plot of rat urine protein after intestine cleaning. E) Scatter plot of rat urinary protein after 1 week of FMT. F) Scatter plot of rat urinary protein after 2 weeks of FMT. G) Scatter plot of rat urinary protein after 3 weeks of FMT. H) HE, PAS, and Masson staining of glomerulus in different groups. I) Electron microscopy showed detailed changes in glomerular basement membrane area in each group. T/Cr, Total urinary protein/urinary creatinine; HE, hematoxylin‐eosin staining; PAS, periodic acid‐schiff staining. *p < 0.05; **p < 0.01; ***p < 0.001; NS, no statistical difference.

Fecal microbiome transplantation (FMT) has been widely used in animal models. The human patient samples indicated a relationship between the gut microbiome and MN. To explore the role of the gut microbiome in the pathogenesis, we established MN model rats. The grouping and process of model construction are shown in **Figure** **6**A. The dynamic changes in the average urine protein in each group are shown using point‐fold line chart in Figure 6B. Scatter plots showed that, at baseline, the groups had no statistically significant differences in urinary protein levels (Figure 6C). Interestingly, the use of antibiotics significantly inhibited the full presentation of the MN phenotype (Figure 6D and Figure S5A,B, Supporting Information; Group Mod+Con, Mod+MN versus Group Model, 8 rats in each group). Furthermore, even in control rats, the use of antibiotics significantly lowered urinary protein levels (Figure 6D, Group Nor+Con, Nor+MN versus Group Normal). This effect quickly disappeared when antibiotics were discontinued (FMT for 1 week, Figure 6D,E and Figure S5B, Supporting Information, Group d‐f). Compared with the Model group, the FMT groups (Groups e and f), either from healthy donors or MN donors, showed delayed and reduced presentation of phenotype (Figure 6B). Contrary to expectations, FMT using MN feces had a little effect on aggravating urinary protein excretion until the animals were euthanized. Interestingly, healthy feces partially replicated the MN model (Figure 6B,E–G, and Figure S5B, Supporting Information). This indicates that a naturally existing microbiome is essential for pathogenesis of MN. The lack of a natural microbiome prevents MN development, whereas restoring it, the MN phenotype can be partially reconstructed.

In terms of histological changes, HE (hematoxylin‐eosin), PAS (periodic Acid‐Schiff), and Masson staining showed significant glomerular damages in Model rats at the end of the study. Consistent with urinary protein, FMT using MN feces showed milder glomerular damage than those using healthy feces (Figure 6H). The electronic microscope showed finder details of the basement membrane, and the results were further verified from the light microscope (Figure 6I). Histological results confirmed that the naturally existing gut microbiome is a prerequisite condition in MN pathogenesis, whereas changes in the gut microbiome in MN patients are more likely to play a protective role in the onset of MN.

### 2.6 Analysis of Rat Fecal Microbiome Confirmed Effective Elimination and Transplantation of Microbe

**Figure 7:**
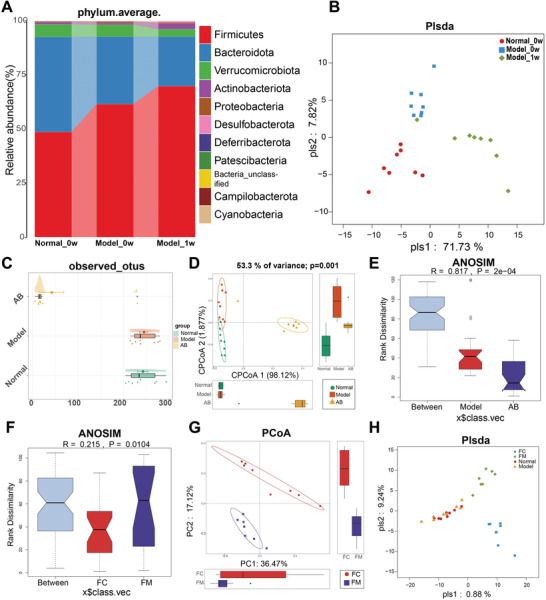
The rat fecal microbiome analysis showed effective elimination and transplantation of microbe. A) Diagram of average compositions and relative abundances of the bacterial communities at phylum level showed the changes were more obvious at Model_1w than Model_0w. B) Plsda axis showed the different compositions at different time points in MN group. C) The cloudplot showed that observed OTUs were markedly decreased after using antibiotics (*p* < 0.001). D) PCoA diagram showed obvious change in gut microbial composition after intestines cleaning (*p* = 0.001). E) Diagram of ANOSIM showed significant difference after using antibiotics (*p* = 0.0002). F) Diagram of ANOSIM showed microbial composition is different after FMT using feces of Cons or MNs for 1 week (*p* = 0.0104). G) Diagram of PCoA showed composition of microbe was significantly different after FMT with feces from Cons or MNs for 1 week. H) Plsda axis showed different microbial compositions in different groups; FC, FMT with feces from Con donor in Mod+Con group; FM, FMT with feces from MN in Mod+MN group; AB, gut microbiome cleaning after using antibiotic.

Fecal samples of rats at different time points were collected and subjected to 16S rRNA sequencing. Since the excretion of urinary protein after model establishment was largest at 1 week, we compared the gut microbiome among three points, which were defined as Normal_0w (fecal collection at 0w from group a), Model_0w (fecal collection at 0w, when the model was established in group d) and Model_1w (fecal sample collection at 1w, 1 week after model establishment in group d). The gut microbiome of MN model rats was altered from that of control rats at 0w. These changes were more significant at 1w, even when the urinary protein excretion diminished (**Figure** **7**A and Figure S6A, Supporting Information), indicating a delay in microbiome adaptation. The composition of the gut microbiome differed among the three groups of fecal samples, as shown in the PLS‐DA diagram and heatmap (Figure 7B and Figure S6B, Supporting Information). Observed OTUs were markedly decreased after using the antibiotic solution (AB, fecal collection at 0w from group e), as shown by cloudplot, PCoA, and ANOSIM (Figure 7C–E and Figure S6C–E, Supporting Information), indicating successful gut microbiota cleansing. The microbial composition of the FMT groups (groups e and f) differed after FMT for 1 week (Figure 7F,G). Each of them differed from the Normal or Model group shown in PLS‐DA diagram, heatmap, and PCoA diagram (Figure 7H and Figure S6F,G, Supporting Information). Both in phylum (Figure S7A,B, Supporting Information) and genus (Figure S7C–G, Supporting Information), relative abundance of gut microbiome showed obvious difference, suggesting successful transplantation of feces. Analyses of rat feces confirmed that the gut microbiome was successfully eliminated and transplanted into different groups. Together with the physiological and pathological results, this study demonstrates that the natural gut microbiome is a crucial factor responsible for MN development.

## 3 Discussion

This multicenter study included 825 fecal samples from both MN patients and healthy controls. The results from this study indicated significant dysbiosis in patients with MN. The diagnostic model based on key OTUs demonstrated excellent identification capability and was validated by various external cohorts. As demonstrated in the rat model, the natural gut microbiome participates in promoting disease onset.

The composition of the gut microbiome changes rapidly from the point of MN onset. Among the genera with obvious changes, *Prevotella* is recognized as beneficial taxa in weight control and glucose metabolism.^[^ ^19^, ^20^ ^]^ *Escherichia−Shigella*, a harmful microbiome for humans, has higher abundance in UMNs. These genera with specific changes may be related to the onset of MN. However, typical changes remain stable even after treatment or disease alleviation. This is different from previous results reported by Ren et al.,^[^ ^13^ ^]^ who demonstrated that the gut microbiome changed gradually with the development of chronic kidney disease. In children with acute lymphoblastic leukemia, schizophrenia, and rheumatoid arthritis, the administration of drugs alters the gut microbial composition.^[^ ^21^, ^22^, ^23^ ^]^ To confirm whether administered medication affected the gut microbiome, we analyzed our data using different methods. However, the pattern of microbiome changed little across different states of MN, irrespective of whether drugs were utilized. The gut microbiome may promptly sense MN onset and adapt itself throughout the course. Another possible reason is that the composition of the microbiome changed significantly compared with that of HCs, and changes between different states of MN were smoothed out.

The gut microbiome has been suggested to be related to an increasing number of diseases in recent years and can even be used as a non‐invasive diagnostic tool for many diseases, such as COVID‐19.^[^ ^11^, ^12^, ^24^ ^]^ In our study, a diagnostic model was successfully constructed using seven crucial OTUs by fivefold cross‐validation using random forest model. The discrimination model showed a good identification ability in both the training and test groups. The subsequent validations using samples from East and South China, and samples with high urinary protein and low urinary protein cohorts also reached more than 90% diagnostic efficiency. The multicenter grouping verification of patients with different disease states suggested a high discrimination capability for all MN patients, thus, providing a new non‐invasive tool for MN diagnosis. The high diagnostic efficacy of the model in different cohorts also indicates that the typical microbiome change was hardly affected by drug treatment or disease status.

Whether the key OTUs are related to MN pathogenesis is unknown. However, the metabolism of these microbes may affect host physiology. According to previous reports, *Lachnospiraceae* was thought to be related to metabolic disturbance, including glucose and/or lipid metabolism.^[^ ^25^, ^26^ ^]^ Similarly, *Agathobacter*, a novel species in the family *Lachnospiraceae*, produces butyrate, acetate, hydrogen, and lactate.^[^ ^27^ ^]^ *Roseburia* and certain species of *Faecalibacterium* can ferment various sugars into short‐chain fatty acids (SCFA).^[^ ^28^, ^29^, ^30^ ^]^ These SCFAs have great benefits to human health, and the abnormal state of SCFAs may lead to diseases.^[^ ^31^, ^32^, ^33^ ^]^ Dysbiosis of the gut microbiome, particularly an increase in pathogenic bacteria and decrease in SCFA‐producing bacteria, may cause or aggravate primary MN. However, the influence of these metabolic pathways on MN still needs to be investigated.

An MN rat model was established to explore the causal relationship between the pathogenesis of MN and alteration of the gut microbiome. The rate of formation of rat model decreased after gut microbiota clearance. This reason might be related to decreased levels of proinflammatory cytokines and fibrosis markers when the gut microbiota is depleted.^[^ ^34^ ^]^ Accordingly, after FMT with fecal samples from HCs or MNs, the urinary protein level showed an obvious increase in gut purged MN rat models. These results may imply that the gut microbiome is necessary for MN onset. Interestingly, 1 week after FMT, rat models transplanted with control microbes had a much more severe disease state than Model group, suggesting that a normal gut microbiome, instead of a disordered gut microbiome, is the precondition for MN. We surmised that the gut flora of MN patients could undergo adaptive changes due to disease onset, helping the host prevent disease progression. This could help explain why the urinary protein level of the Mod+MN group was the lowest among the three MN rat groups. Our study mainly focused on the onset mechanism of MN. As for the treatment effect of FMT in MN, more studies will be needed. A recent case reported a patient of MN got remission after FMT using fecal microbiota from a healthy donor.^[^ ^35^ ^]^ We believe a rigorously designed controlled clinical study will help to confirm the result. What's more, metagenomic sequencing will be needed to find key stains that play roles in disease onset and treatment. Another interesting phenomenon was the unparalleled clinical manifestation and gut dysbiosis. Although the Model group had severe clinical symptoms at 0 week, the microbial differences were more significant at 1 week after FMT. A similar phenomenon has also been observed in hepatitis B virus‐infected mice^[^ ^36^ ^]^ This might be due to the relatively delayed changes in the gut microbiome compared with alterations in clinical symptoms. Once again, this gave credence to the conjecture that the gut flora was an adaptive change in MN.

Our study had several advantages. First, the sample size was relatively large. Second, the diagnostic model based on the gut microbiome provides a non‐invasive and broadly applicable tool for clinically diagnosing MN. Most importantly, we proved that a naturally existing gut microbiome is essential for MN pathogenesis, which could be developed as a novel therapy in the future. Our study also had some limitations. The molecular mechanisms by which the gut microbiome affects MN pathogenesis remain unknown. The difference in the gut microbiome between species is another point that should not be neglected, therefore, the results of the rat model need to be clinically validated in humans.

## 4 Conclusion

The gut microbiome of patients with MN varies greatly from that of healthy individuals. Gut microbiome analysis could be used to non ‐invasively diagnose MN. Moreover, the onset of MN depends on the existence of the natural gut microbiome, and subsequent changes in the gut microbiome in MN patients can play a protective role. These findings may be used to provide novel targets for preventing and diagnosing MN.

## 5 Experimental Section

### Study Cohort

From April 2019 to January 2020, a total of 966 fecal samples from MN patients and healthy controls were collected from three medical centers in Central China (The First Affiliated Hospital of Zhengzhou University), East China (Shandong Provincial Hospital), and South China (The Third Affiliated Hospital of Sun Yat‐sen University). All MN cases were pathologically diagnosed as primary MN. The exclusion criteria were as follows: 1) application of antibiotics within 3 months before enrollment; 2) obvious infection, such as oral, respiratory, and digestive tract; 3) co‐morbidity of other diseases such as tumor, digestive system disease, lupus, and diabetes; 4) complete remission of MN (stable renal function and urine protein < 0.3 g/24 h), and 5) repeated or incomplete information. Finally, 825 fecal samples were included for further research including 370 samples of MN patients from the First Affiliated Hospital of Zhengzhou University (stored in the Biobank of The First Affiliated Hospital of Zhengzhou University and National Human Genetic Resources Sharing Service Platform, Grant No. 2005DKA21300), 65 samples of MN patients from Shandong Provincial Hospital, 44 samples of UMN patients from the Third Affiliated Hospital of Sun Yat‐sen University, and 346 samples of healthy controls from Physical Examination Center of the First Affiliated Hospital of Zhengzhou University. None of the healthy controls had taken antibiotics, probiotics, or prebiotics for at least 3 months prior to sample collection.

Among 370 patients with MN from Central China, 115 were not administered medication before sample collection. These were defined as UMN. The remaining 255 patients who had taken immunosuppressants or corticosteroids were defined as treated MNs (TMNs). TMNs were further divided into HPRO group (urinary protein ≧3.5 g/24 h, *n* = 84) and LPRO (urinary protein < 3.5 g/24 h, *n* = 171). All the baseline data were obtained at the time of stool sample collection.

### Stool Sample Collection and DNA Extraction

All stool samples collected from patients with MN and healthy volunteers were immediately stored at 4 °C. They were then transferred to −80 °C environment within 2 h for further analysis. DNA extraction was performed using the E.Z.N.A. Stool DNA Kit. All procedures were performed by Shanghai Mobio Biomedical Technology Co. Ltd. using the MiSeq platform (Illumina Inc., USA) according to the manufacturer's protocols. The primers F1 and R2 (5′‐ CCTACGGGNGGCWGCAG‐3′ and 5′‐GACTACHVGGGTATCTAATCC‐3′) were used to amplify the V3 ≈ V4 region of each fecal sample by PCR. Detailed DNA extraction steps were performed as previously described,^[^ ^37^ ^]^ and are shown in Document 1, Supporting Information.

### OTU Clustering, Comparison of Gut Microbiome, and Diagnostic Model Construction

After 16S rRNA sequencing, OTUs were classified based on 97% similarity and the RDP Classifier was used (http://rdp.cme.msu.edu/) against the Silva (SSU138) 16S rRNA database with a confidence threshold of 70% to analyze the phylogenetic affiliation of the 16S rRNA gene sequence.^[^ ^38^ ^]^ The composition, diversity, and alterations in the gut microbiome were compared and functional changes in patients were predicted with MN. Microbial diversity was presented using ACE index, Shannon index, Simpson index, and Chao index. Microbial composition at the phylum and genus levels was compared. PCoA was used to represent the microbiome distribution between samples. The linear discriminant analysis effect size (LEfSe) method was used to characterize fecal microbiota.^[^ ^39^ ^]^ Detailed statistical analysis and tests are provided shown in Document 2, Supporting Information.

The participants of the UMNs and matched HCs were randomly divided into training and test groups. Based on the abundance profiles of OTU, fivefold cross‐validation of random forest model was used to feature important OTUs (importance value > 0.001) and the POD for each individual was calculated. Subsequently, as previously described,^[^ ^40^ ^]^ a ROC curve was drawn and the AUC was used to evaluate the diagnostic efficacy of the gut microbiome in UMNs and HCs.

### Verification of the Efficiency and the Application Range of the Diagnostic Model

After establishing the diagnostic model, the test group was used as the internal validation cohort to verify the efficiency of the model. First‐onset MN patients from South China and matched HCs were used as cross‐regional validation cohorts to evaluate the model. The diagnostic efficiency of the model was validated in 255 patients with TMN and 205 matched HCs. 65 patients with MN from Shandong and matched HCs were also included in the diagnostic model for cross‐regional validation. Finally, 84 HPRO and 171 LPRO patients, along with their HCs, were included in the model to explore the link between disease states and the diagnostic model.

### Establishment of Rat Model

For this study 48 male Sprague Dawley (SD) rats weighing 80–100 g were obtained from Spaefer Biotechnology Co., Ltd. All rats were fed under a 12 h light/dark cycle at 22–24 °C. Standard diets and drinking water were provided. The rats were randomly divided into six groups and there were 8 rats in each group: “a” (Normal) normal rat group, “b” (Nor+Con) normal rats with FMT from healthy donors, “c” (Nor+MN) normal rats with FMT from MN donors, “d” (Model) MN model group, “e” (Mod+Con) MN rats with FMT from healthy donors, “f” (Mod+MN) MN rats with FMT from MN donors. All rats received an adaptive feeding for 1 week.

Gut microbiota cleansing and FMT were used to explore the relationship between the gut microbiome and MN. The antimicrobial solution consisted of ampicillin 0.1 g L^−1^, vancomycin 0.5 g L^−1^, neomycin 1 g L^−1^, and metronidazole 1 g L^−1^, which replaced the purified water for daily water intake. The antimicrobial solution was changed every 2 days. Stool samples from four MN patients and four age‐and‐sex‐matched HCs were mixed‐up to prepare bacterial solutions for FMT. The preparations of fecal bacterial solutions from healthy donors and MN donors are shown in Document 3, Supporting Information.

Sheep anti‐Rat Fx1A serum (PTX‐002S) was used to induce the passive Heymann nephritis (PHN) in SD rats.^[^ ^41^ ^]^ Rats in group d (Model group), e (Mod+Con), and f (Mod+MN) were injected with 0.4 mL/100 mg body weight of anti‐Fx1A serum into tail vein once a day for 2 weeks. After starting injection for 3 days, the rats in groups b (Nor+Con), c (Nor+MN), e (Mod+Con), and f (Mod+MN) were then subjected to a 14‐day course of antimicrobial solution for gut microbiome clearance with antimicrobial solution instead of drinking water.

After gut microbiome clearance and PTX‐002S injection, the rat models in groups b (Nor+Con) and e (Mod+Con) were subjected to FMT by the bacterial solution from healthy donors (1 mL day^−1^) for 21 days, and equal amounts of bacterial solution from MN donors were also administered into rat models in groups c (Nor+MN) and f (Mod+MN) for 21 days. The feces and urine of each rat were collected once per week until they were euthanized after 21 days of FMT. The detailed modeling process and sample collection for MN rats are shown in Document 4, Supporting Information.

### Clinical Features, Pathological Changes, and Gut Microbiome Alterations in Rat Model

The total urinary protein/urinary creatinine (T/Cr) ratio was used to estimate the severity of MN. Hematoxylin‐eosin (HE), periodic acid‐Schiff (PAS), and Masson staining were used to observe glomerular histomorphology. The detailed staining process is described in Document 5, Supporting Information. A transparent electron microscope (Japan Electronics Co., Ltd) was used to observe the area of the glomerular basement membrane. The fecal samples collected from the rat models were subjected to 16S rRNA sequencing to explore alterations in the gut microbiome in different groups and at different time points.

### Statistical Analysis

Categorical data were presented as percentages. All the continuous variables were tested for normality. Mean and standard deviation (SD) was described as continuous variables satisfying the normal distribution, while medians (quartile 1, quartile 3) were used to present the variables that did not satisfy the normal distribution. The *t*‐test for normal continuous variables, Wilcoxon rank‐sum test for non‐normal continuous variables, and Chi‐square test or Fisher's exact test for categorical variables were used to compare the differences between different groups. Statistical significance was set at *P* < 0.05. The statistical analyses were completed using R software, version 4.0.2 (http://www.R‐project.org/), and SPSS, version 24.0.

### Availability of Data and Materials

Raw Illumina read data for all samples were deposited in the European Bioinformatics Institute European Nucleotide Archive database (PRJNA752445).

### Ethics Approval and Consent to Participate

The First Affiliated Hospital of Zhengzhou University Ethics Review Committee granted ethical approval for the study and the ethics review approval ID is “2019‐KY‐361.” The ethics review approval ID in Shandong Provincial Hospital is “SWYX: NO. 2020‐077.” All the participants included in this project provided written informed consents. The ethics review approval ID of animal experiments is “ZZU‐LAC20210115[02].”

## Conflict of Interest

The authors declare no conflict of interest.

## Author Contributions

J.S., Y.Z., R.G., and W.L. contributed equally to this work. J.S., J.X., and Z.Z. designed the research and experiments. Y.Z. and R.G. wrote the manuscript. W.L. and Jun Z. collected the samples. G.Y., F.W., W.C., P.W., X.Z., and T.W. finished the experiments. Y.D., Jing Z., and L.W. participated in data statistical analysis. H.S. reviewed and modified the manuscript. All authors participated in the revision of the manuscript.

## Supporting information

Supporting Information

## Data Availability

The data that support the findings of this study are openly available in European Bioinformatics Institute European Nucleotide Archive database at https://www.ebi.ac.uk/ena/browser/advanced‐search, reference number 752445.
