# Supplementary material for: Gut Microbiome Analysis Can Be Used as a Noninvasive Diagnostic Tool and Plays an Essential Role in the Onset of Membranous Nephropathy
Source: Adv Sci (Weinh). 2022 Aug 17;9(28):2201581. doi: 10.1002/advs.202201581 (PMC9534961; doi:10.1002/advs.202201581)
Supplement: Supplementary file 1 — Supporting Information [file ADVS-9-2201581-s001.pdf]

ADVANCED SCIENCE
Open Access

# Supporting Information

for *Adv. Sci.*, DOI 10.1002/advs.202201581

Gut Microbiome Analysis Can Be Used as a Noninvasive Diagnostic Tool and Plays an Essential Role in the Onset of Membranous Nephropathy

*Jin Shang*, Yiding Zhang, Ruixue Guo, Wenli Liu, Jun Zhang, Ge Yan, Feng Wu, Wen Cui, Peipei Wang, Xuejun Zheng, Ting Wang, Yijun Dong, Jing Zhao, Li Wang, Jing Xiao and Zhanzheng Zhao**

Table S1. The baseline characteristics of 115 UMNs and 115 age-and-gender matched HCs. HC, healthy control; UMN, untreated membranous nephropathy; Scr, serum creatinine; ALB, albumin; CHO, cholesterol; TG, Triglyceride; PLA2R, phospholipase A2 receptor, PLA2R; THSD7A, thrombospondin type-1 domain-containing 7A.

| | HC n=115 | UMN n=115 | P value |
|---|---|---|---|
| Gender | | | 1.000 |
| male | 60 (52.2%) | 61 (53.0%) | |
| female | 55 (47.8%) | 54 (47.0%) | |
| Age(years) | 48.0 [40.5;55.0] | 51.0 [41.5;56.0] | 0.356 |
| Urine protein (g) | 0.08 [0.06;0.09] | 4.80 [3.17;7.86] | <0.001 |
| Scr (μmol/L) | 67.0 [59.0;78.0] | 69.0 [56.0;84.0] | 0.466 |
| ALB (g/L) | 48.0 [46.0;49.4] | 25.2 [20.4;30.6] | <0.001 |
| CHO (mmol/L) | 4.78 [4.16;5.23] | 6.01 [4.93;7.62] | <0.001 |
| TG (mmol/L) | 1.16 [0.90;1.48] | 1.81 [1.25;2.83] | <0.001 |
| | | N=111 | |
| PLAR (RU/mL) | | | |
| <14 | | 37 （33.3%) | |
| >=14 | | 74 （66.7%） | |
| THSD7A: | | | |
| <100 | | 103 (92.8%) | |
| >=100 | | 8 (7.2%) | |

Table S2. The baseline characteristics of discovery cohort (UMN_discovery=72, HC_discovery=72).

| | HC_discovery n=72 | UMN_discovery n=72 | P value |
|---|---|---|---|
| Gender | | | 0.182 |
| male | 33 (45.8%) | 41 (56.9%) | |
| female | 39 (54.2%) | 31 (43.1%) | |
| Age(years) | 48.99±8.98 | 48.74±11.72 | 0.739 |
| Urine protein (g) | 0.08 (0.06, 0.10) | 4.84 (3.37, 8.03) | <0.01 |
| Scr (μmol/L) | 65.0 (59.0, 76.8) | 68.5 (56.3, 84.0) | <0.01 |
| ALB (g/L) | 48.0 (45.5, 49.2) | 25.6±6.8 | 0.309 |
| CHO (mmol/L) | 4.69±0.73 | 6.34±1.84 | <0.01 |
| TG (mmol/L) | 1.08 (0.89, 1.45) | 1.95 (1.32, 3.10) | <0.01 |
| | | N=69 | |
| PLAR (RU/mL) | | | |
| <14 | | 24 (34.8%) | |
| >=14 | | 45 (65.2% ) | |
| THSD7A: | | | |
| <100 | | 63 (91.3%) | |
| >=100 | | 6 (8.7%) | |

Table S3. The baseline characteristics of validation cohort (UMN_validation=43, HC_discovery=43).

| | HC_validation n=43 | UMN_validition n=43 | P value |
|---|---|---|---|
| Gender | | | 0.129 |
| male | 27 (62.8) | 20 (46.5%) | |

| | | | |
|---|---|---|---|
| female | 16 (37.2) | 23 (53.5%) | |
| Age(years) | 48.00 (38.00, 55.00) | 49.51±11.83 | 0.36 |
| Urine protein (g) | 0.08 (0.06, 0.09) | 5.18±3.21 | <0.01 |
| Scr (μmol/L) | 69.42±11.85 | 72.35±22.30 | 0.935 |
| ALB (g/L) | 48.1±9.06 | 25.5±7.11 | <0.01 |
| CHO (mmol/L) | 4.71±0.66 | 5.91 (4.91, 7.68) | <0.01 |
| TG (mmol/L) | 1.23±0.39 | 1.63 (1.16, 2.28) | <0.01 |
| | | N=42 | |
| PLAR (RU/mL) | | | |
| <14 | | 13 (31.0%) | |
| >=14 | | 29 (69.0%） | |
| THSD7A: | | | |
| <100 | | 40 (95.0%) | |
| >=100 | | 2 (5.0%) | |

Table S4. Clinical data of 78 UMNs, 108 TMNs and 100 HCs. TMN, treated membranous nephropathy.

| | HC N=100 | TMN N=108 | UMN N=78 | p.overall | p.UMN vs HC | p.UMN vs TMN | p.HC vs TMN |
|---|---|---|---|---|---|---|---|
| Gender: | | | | 0.688 | 0.799 | 0.799 | 0.799 |
| female | 41 (41.0%) | 48 (44.4%) | 37 (47.4%) | | | | |
| male | 59 (59.0%) | 60 (55.6%) | 41 (52.6%) | | | | |

| | | | | | | | |
|---|---|---|---|---|---|---|---|
| Age(years) | 48.0 [42.8;53.0] | 50.0 [40.0;55.0] | 49.0 [40.2;55.8] | 0.966 | 0.988 | 0.988 | 0.988 |
| Urine protein (g) | 0.08 [0.06;0.10] | 3.72 [1.87;6.24] | 4.35 [3.07;6.74] | <0.001 | <0.001 | 0.053 | <0.001 |
| Scr (μmol/L) | 69.5 [60.8;77.0] | 72.5 [61.8;82.0] | 68.0 [56.0;85.0] | 0.263 | 0.266 | 0.611 | 0.646 |
| ALB (g/L) | 47.9 [46.0;49.0] | 27.8 [21.0;32.6] | 26.7 [23.2;31.6] | <0.001 | <0.001 | 0.809 | <0.001 |
| CHO (mmol/L) | 4.59 [4.12;5.04] | 5.58 [4.66;6.89] | 5.92 [4.83;7.50] | <0.001 | <0.001 | 0.344 | <0.001 |
| TG (mmol/L) | 1.15 [0.89;1.46] | 1.89 [1.35;2.80] | 1.74 [1.23;2.81] | <0.001 | <0.001 | 0.447 | <0.001 |
| PLA (RU/mL) | | 10.8 [2.00;44.3] | 19.1 [3.05;65.9] | 0.089 | | | |
| TSH7A | | 30.6 [20.8;41.1] | 25.1 [16.8;42.5] | 0.135 | | | |

Table S5. Comparison of clinical data in HPROs and LPROs. HPRO, high urine protein; LPRO, low urine protein.

| | HPro N=84 | LPro N=171 | p.HPro vs LPro |
|---|---|---|---|
| Gender: | | | 0.819 |
| female | 28 (33.3%) | 61 (35.7%) | |
| male | 56 (66.7%) | 110 (64.3%) | |
| Age(years) | 48.5 [36.0;54.2] | 47.0 [37.0;55.0] | 0.864 |
| Urine protein (g) | 6.02 [4.67;7.56] | 1.04 [0.58;1.98] | <0.001 |
| Scr (μmol/L) | 76.0 [66.1;100] | 74.0 [61.0;87.0] | 0.122 |
| ALB (g/L) | 22.2 [19.0;27.6] | 33.9 [29.4;37.1] | <0.001 |
| CHO (mmol/L) | 6.25 [5.08;7.14] | 4.57 [3.80;5.49] | <0.001 |

| | | | |
|---|---|---|---|
| TG (mmol/L) | 1.93 [1.45;3.09] | 1.58 [1.12;2.25] | <0.001 |
| PLA (RU/mL) | 15.8 [2.60;63.1] | 2.70 [2.00;12.2] | |
| TSH7A | 30.5 [20.0;49.4] | 28.7 [20.0;42.8] | |

Table S6: Clinical data of discovery phase in HPROs and LPROs.

| | HPro N=56 | LPro N=114 | p-value |
|---|---|---|---|
| Gender: | | | 0.404 |
| female | 17 (30.4%) | 42 (36.2%) | |
| male | 39 (69.6%) | 72 (63.8%) | |
| Age(years) | 45.64±13.14 | 48.5 (35.25, 54) | 0.585 |
| Urine protein (g) | 6.02 (4.63, 7.21) | 1.01 (0.58, 1.96) | <0.001 |
| Scr (μmol/L) | 76.0 (67.5, 102.8) | 71.5 (60.0, 89.0) | 0.118 |
| ALB (g/L) | 22.1 (18.7, 28.3) | 33.1±6.3 | <0.001 |
| CHO (mmol/L) | 6.33 (5.06, 8.12) | 4.63 (3.82, 5.56) | <0.001 |
| TG (mmol/L) | 1.79 (1.43, 2.76) | 1.69 (1.18, 2.32) | 0.053 |
| PLA (RU/mL) | 20.8 (2.8, 61.0) | 2.8 (2.0, 12.5) | |
| TSH7A | 31.2 (20.1, 53.2) | 28.7 (20.6, 42.9) | |

Table S7: Clinical data of validation phase in HPROs and LPROs.

| | HPro N=28 | LPro N=57 | p-value |
|---|---|---|---|
| Gender: | | | 0.291 |
| female | 11 (28.2%) | 19 (25%) | |

| | | | |
|---|---|---|---|
| male | 28 (71.8) | 57 (75%) | |
| Age(years) | 47.32±13.13 | 46.28±12.80 | 0.64 |
| Urine protein (g) | 6.01 (4.78, 7.87) | 1.15 (0.56, 2.12) | <0.001 |
| Scr (μmol/L) | 78.0 (65.5, 102.8) | 76.23±17.16 | 0.575 |
| ALB (g/L) | 22.5 (19.3, 27.5) | 32.8±6.00 | <0.001 |
| CHO (mmol/L) | 6.27±2.11 | 4.45 (3.75, 5.39) | 0.001 |
| TG (mmol/L) | 2.22 (1.48, 3.79) | 1.40 (1.01, 1.98) | 0.001 |
| PLA (RU/mL) | 11.7 (2.0 80.0) | 2.1 (2.0, 12.3) | |
| TSH7A | 29.7 (19.6, 41.8) | 28.7 (20.0, 42.8) | |

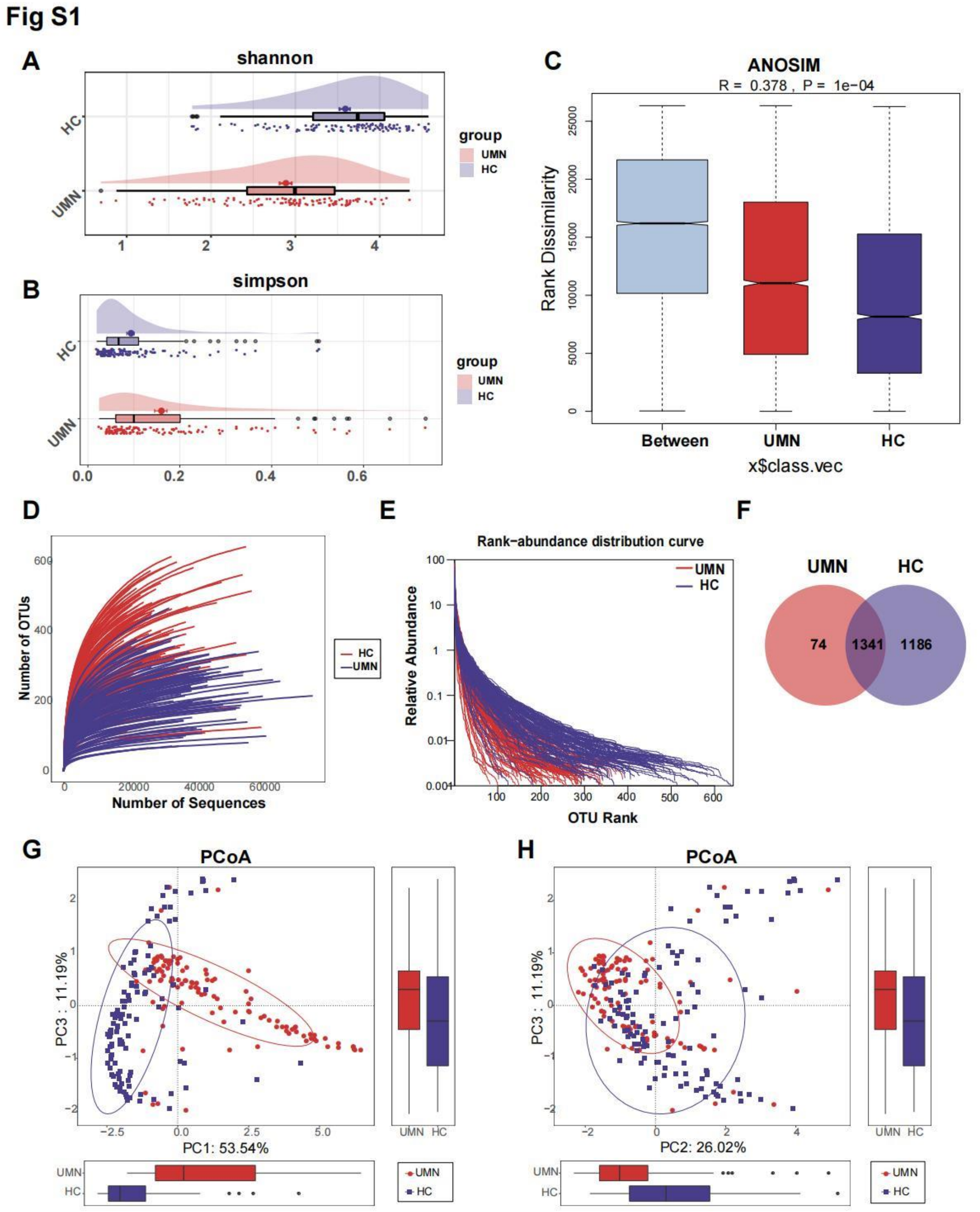


Figure S1. The supplement to diversity of microbiota between UMNs (n=115) and HCs (n=115). (A) Cloudplot showed α-diversity of Shannon index in UMNs and HCs ($p<0.001$). (B) Cloudplot showed α-diversity of Simpson index in UMNs and HCs ($p<0.001$). (C) ANOSIM showed the difference between groups was greater than that within groups in UMN group and HC group ($p<0.0001$). (D) Rarefaction curves showed HCs had more observed OTUs than UMNs in general. (E) The abundance of OTU rank was generally higher in HCs than in UMNs. (F) Venn diagram showed

OTU distribution in UMNs and HCs. (G) PCoA analysis showed visualized β-diversity by unweighted UniFrac algorithm along PC1 and PC3. (H) PCoA analysis showed visualized β-diversity by unweighted UniFrac algorithm along PC2 and PC3. * $p<0.05$; ** $p<0.01$, *** $p<0.001$.

Fig S2

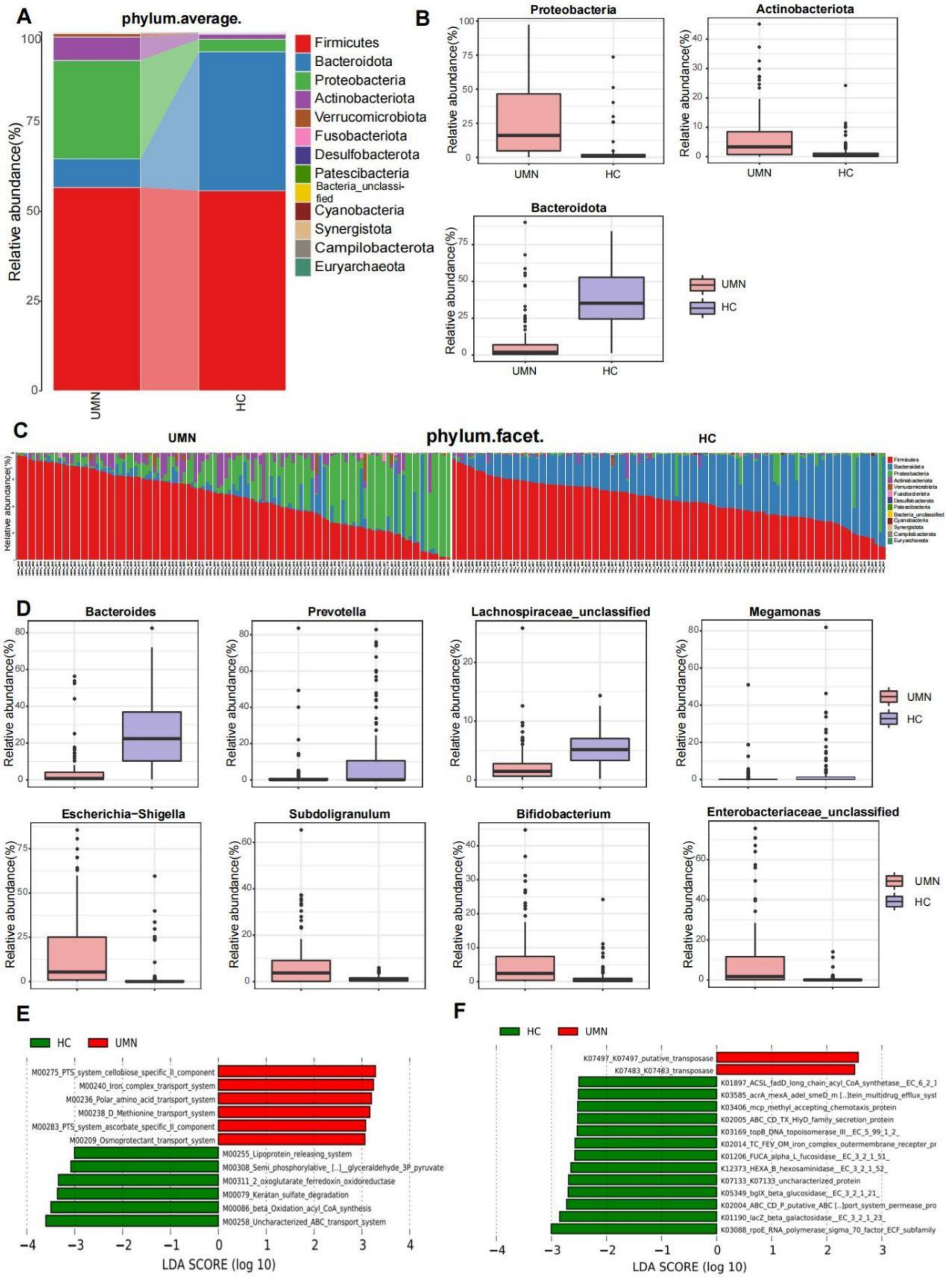


Figure S2. The supplement to characteristics and functional alteration of microbiota between UMNs (n=115) and HCs (n=115). (A) Average compositions and relative abundances of the bacterial communities in UMNs and HCs at phylum level. (B)

Barplot showed the difference in relative abundance of *Proteobacteria*, *Actinobacteria*, and *Bacteroidota* at phylum level. (C) Barplot showed relative abundance of gut microbiota of each sample in two groups at phylum level. (D) Eight genera with high abundance and obvious changes in two groups. (E) Predicted metabolic functions with LDA score >3.0 were obtained by PICRUSt on KEGG. (F) Predicted metabolic pathways with LDA score >3.0 were obtained by PICRUSt on KO. PICRUSt, Phylogenetic Investigation of Communities by Reconstruction of Unobserved States; KEGG, Kyoto Encyclopedia of Genes and Genomes; KO, KEGG orthologous groups; LDA: Linear Discriminant Analysis.

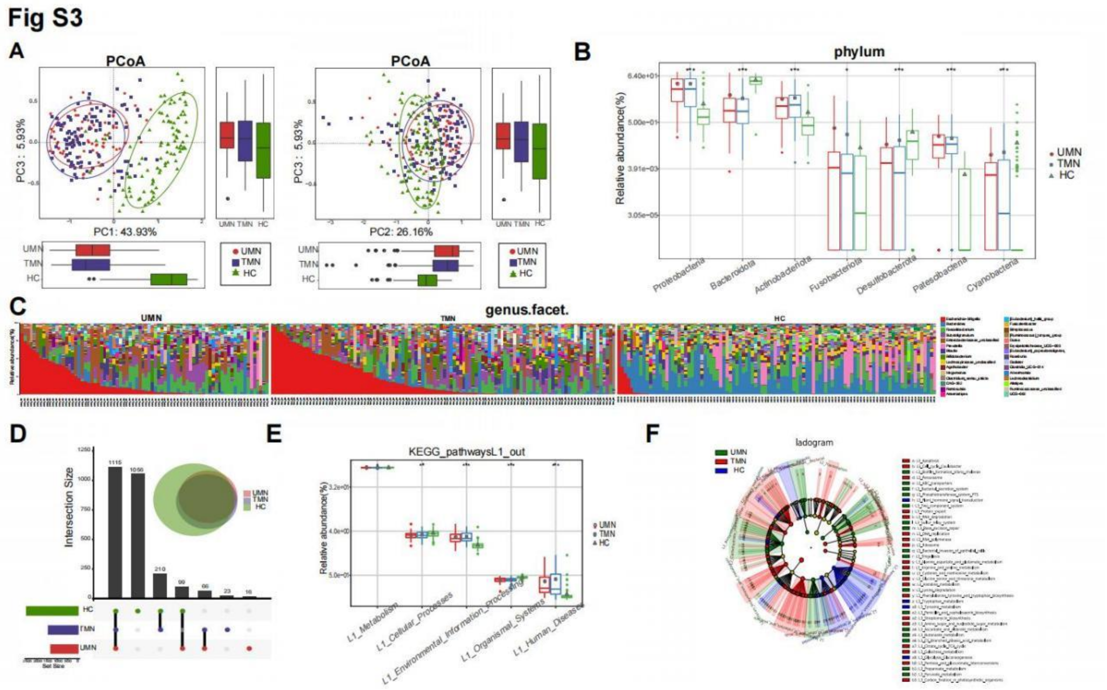


Figure S3. The supplement to characteristics and function alteration of microbiota among UMNs (n=78), TMNs (n=108) and HCs (n=100). (A) PCoA analysis showed visualized β-diversity by unweighted UniFrac algorithm anlong PC1 vs. PC3, PC2 vs. PC3. (B) Barplot showed the difference in relative abundance at phylum level by Wilcoxon Rank test among three groups. (C) Barplot showed average compositions and relative abundances of the bacterial communities from three different groups at genus level (Only the 30 genera with the highest relative abundance were listed). (D) Venn diagram showed OTU distribution in three groups. (E) Comparision of predicted metabolic functions obtained by PICRUSt on KEGG at level 1. (F)

Predicted metabolic pathways with LDA score >3.0 were obtained by PICRUSt on KO at level 2 and level 3. * p<0.05; **p<0.01, ***p<0.001.

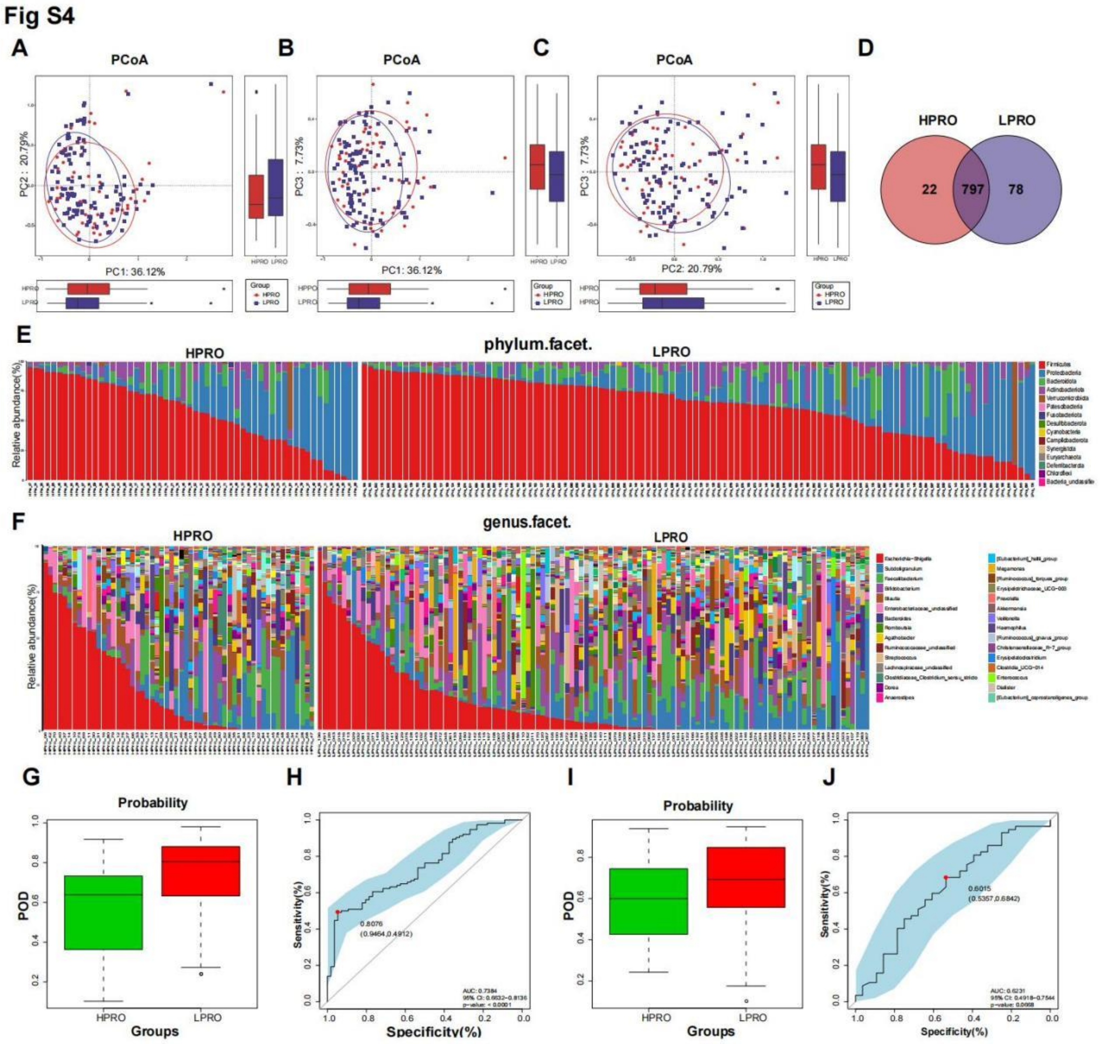


Figure S4. The supplement to characteristics and functional alteration of microbiota between HPROs (n=56) and LPROs (n=114). (A) PCoA analysis showed visualized β-diversity by unweighted UniFrac algorithm along PC1 and PC2. (B) PCoA analysis showed visualized β-diversity by unweighted UniFrac algorithm along PC1 and PC3. (C) PCoA analysis showed visualized β-diversity by unweighted UniFrac algorithm along PC2 and PC3. (D) Venn diagram showed OTU distribution in HPROs and LPROs. (E) Barplot showed relative abundance of gut microbiota from HPROs and LPROs groups at phylum level. (F) Relative abundance of gut microbiome in HPRO

and LPROs groups at genus level (Only the 30 genera with the highest relative abundance were listed). (G) New model was constructed for discrimination of HPROs and LPROs. Comparison of POD value based on 8 microbial markers in the training group of the new model. (H) ROC curve based on 8 obtained microbial markers showed discrimination rate of 73.84% in training group. (I) Comparison of POD value based on 8 microbial markers in the test group. (J) ROC curve showed discrimination rate of 62.31% in test group.

**Fig S5**

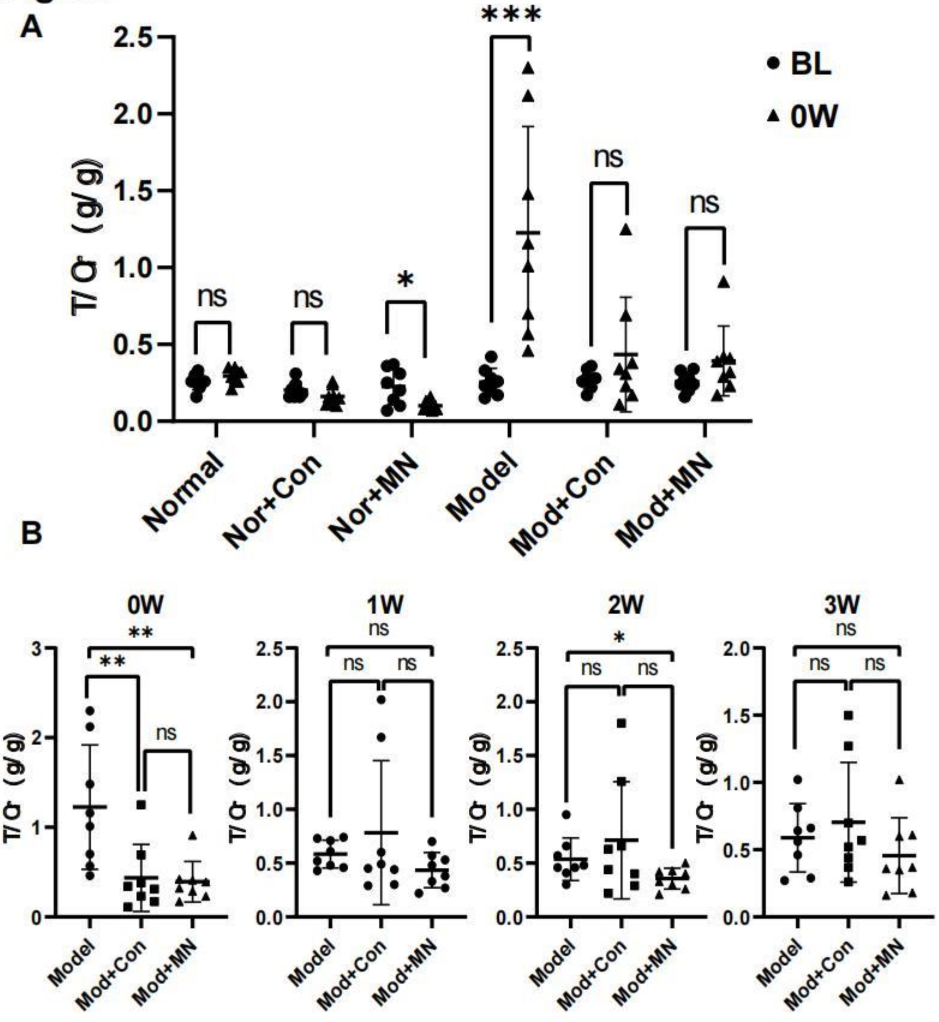

Figure S5. Detailed supplement to the statistical analysis of urinary protein in rat model. (A) Scatter plot showed differences of urinary protein before and after gut microbial cleaning in each group. (B) Scatter plot showed differences of urinary protein from week 0 to week 3 among model, Mod+Con and Mod+MN groups. BL, baseline; T/Cr, Total urinary protein/ urinary creatinine. *p<0.05; **p<0.01; ***p<0.001; ns, no statistical difference.

**Fig S6**

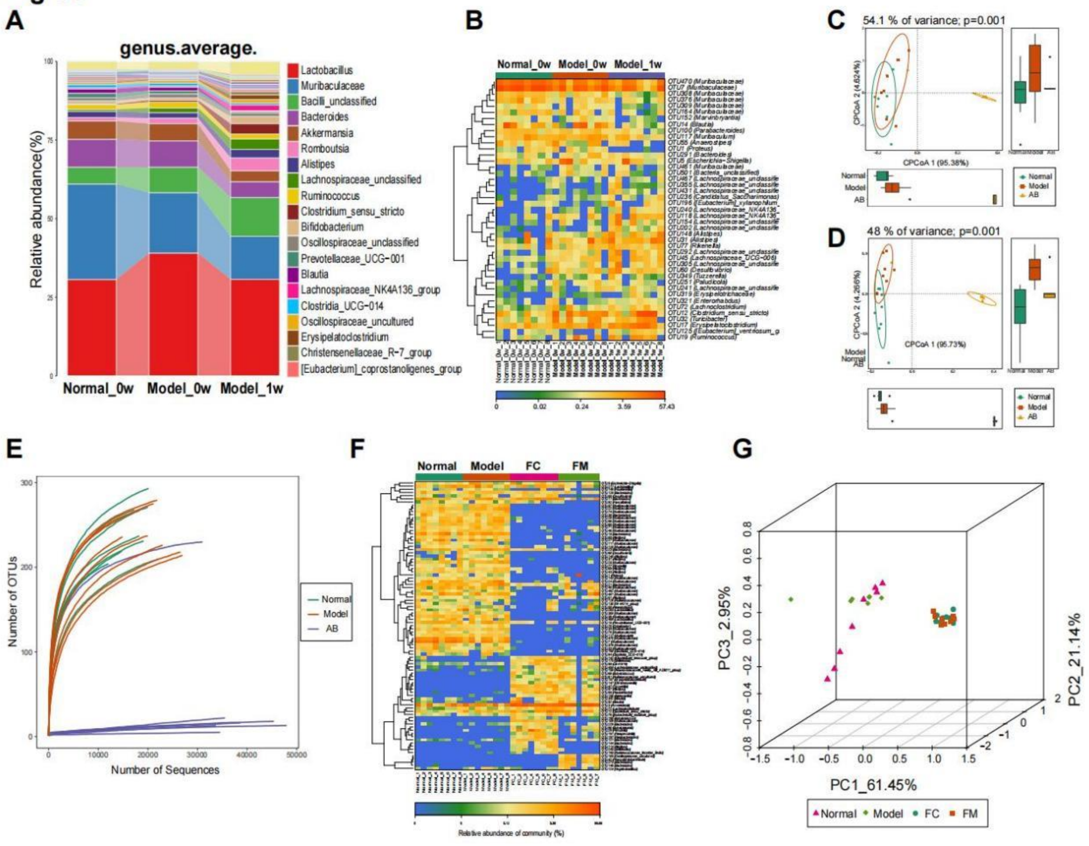


Figure S6. The supplement to characteristics of microbiota in rat model. (A) The relative abundance suggested the alteration of microbial abundance was more obvious at AM than ME at genus level (Only the 20 genera with the highest relative abundance were listed). (B) Heatmap showed the relative abundance of top 1% most

abundant OTUs selected by random forest model in three groups. (C) PCoA analysis of unweighted unifrac showed visualized microbial difference after using antibiotics along PC1 and PC2. (D) PCoA analysis of bray curtis showed visualized microbial difference after using antibiotics along PC1 and PC2. (E) Rarefaction curves showed the decrease of observed OTUs after using antibiotics, suggesting the success of intestine cleaning. (F) Heatmap showed relative abundance of top 1% most abundant OTUs in four groups. (G) Diagram of PCoA suggested different composition of gut microbiome in each group.

**Fig S7**

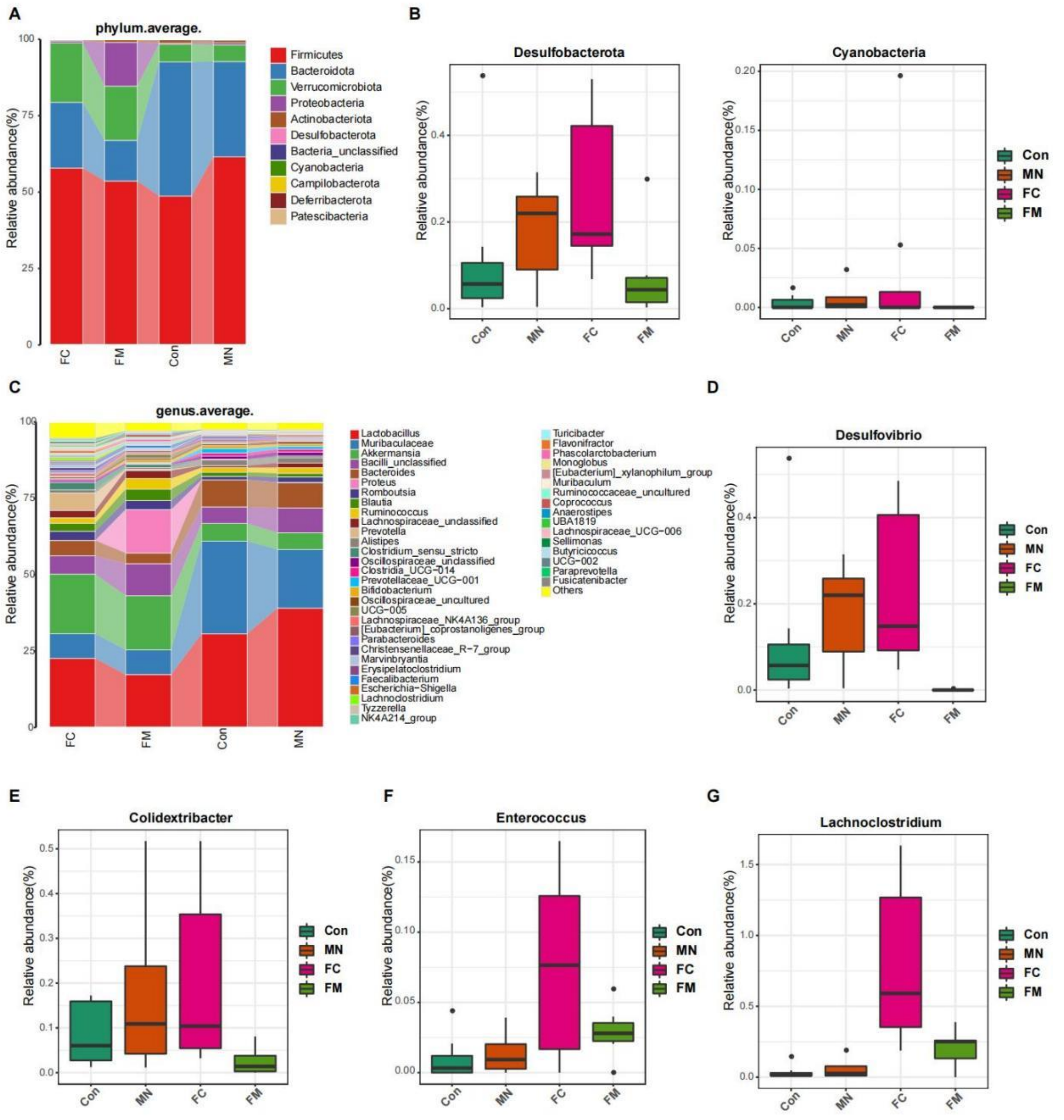

Figure S7. Gut microbiome composition in phylum and genus levels among four different groups. (A) The relative abundance of gut microbiome in phylum level. (B) Comparison of relative abundance of *Desulfobacterota* and *Cyanobacteria* in phylum level among four groups. (C) The relative abundance of gut microbiome in genus level. (D-G) Comparison of relative abundance of *Desulfovibrio* (D), *Colidextribacter* (E), *Enterococcus* (F), and *Lachnoclostridium* (G) in genus level.
